# Cyclohexanedodecol-Assisted Interfacial Engineering for Robust and High-Performance Zinc Metal Anode

**DOI:** 10.1007/s40820-022-00846-0

**Published:** 2022-04-19

**Authors:** Zhenzhen Wu, Meng Li, Yuhui Tian, Hao Chen, Shao-Jian Zhang, Chuang Sun, Chengpeng Li, Milton Kiefel, Chao Lai, Zhan Lin, Shanqing Zhang

**Affiliations:** 1 Centre for Clean Environment and Energy, School of Environment and Science, Griffith University, Gold Coast, 4222 Australia; 2 Guangzhou Key Laboratory of Clean Transportation Energy Chemistry, School of Chemical Engineering and Light Industry, Guangdong University of Technology, Guangzhou, 510006 People’s Republic of China; 3 School of Chemistry and Materials Science, Jiangsu Normal University, Xuzhou, 221116 People’s Republic of China; 4 Institute for Glycomics, Griffith University, Gold Coast, 4222 Australia

**Keywords:** Cyclohexanedodecol, Aqueous Zn-ion battery, Zn dendrite, Zn corrosion, Hydrogen evolution

## Abstract

Aqueous zinc-ion batteries (AZIBs) can be one of the most promising electrochemical energy storage devices for being non-flammable, low-cost, and sustainable. However, the challenges of AZIBs, including dendrite growth, hydrogen evolution, corrosion, and passivation of zinc anode during charging and discharging processes, must be overcome to achieve high cycling performance and stability in practical applications. In this work, we utilize a dual-functional organic additive cyclohexanedodecol (CHD) to firstly establish [Zn(H_2_O)_5_(CHD)]^2+^ complex ion in an aqueous Zn electrolyte and secondly build a robust protection layer on the Zn surface to overcome these dilemmas. Systematic experiments and theoretical calculations are carried out to interpret the working mechanism of CHD. At a very low concentration of 0.1 mg mL^−1^ CHD, long-term reversible Zn plating/stripping could be achieved up to 2200 h at 2 mA cm^−2^, 1000 h at 5 mA cm^−2^, and 650 h at 10 mA cm^−2^ at the fixed capacity of 1 mAh cm^−2^. When matched with V_2_O_5_ cathode, the resultant AZIBs full cell with the CHD-modified electrolyte presents a high capacity of 175 mAh g^−1^ with the capacity retention of 92% after 2000 cycles under 2 A g^−1^. Such a performance could enable the commercialization of AZIBs for applications in grid energy storage and industrial energy storage.

**Supplementary Information:**

The online version contains supplementary material available at 10.1007/s40820-022-00846-0.

## Highlights

- Cyclohexanedodecol (CHD) could facilitate the Zn dendrite-free plating/stripping at a nanoscale.
- The CHD molecules could effectively modify the hydrated Zn(H_2_O)_6_^2+^ structure in aqueous Zn ion batteries.
- The addition of CHD could establish robust protection layers on the Zn electrode surface.
- The CHD-modified electrolytes exhibit long-term cycling stability.

**Supplementary Information:**

The online version contains supplementary material available at 10.1007/s40820-022-00846-0.

## Graphical abstract

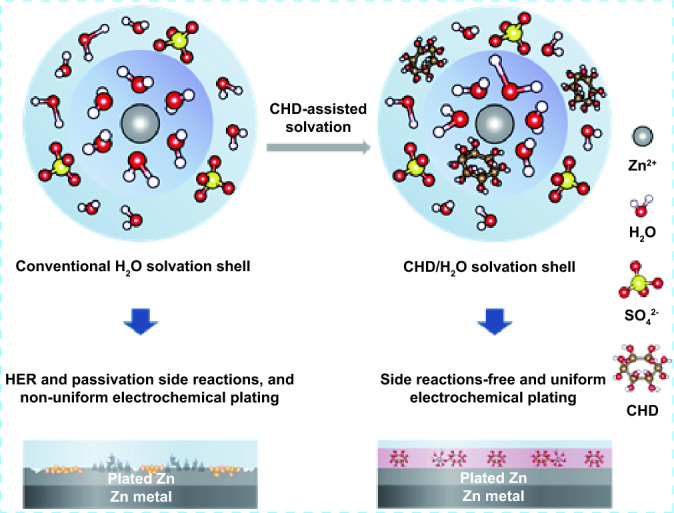

## Introduction

Besides the revolution of the electroactive materials, the development of electrolytes has been contributing an essential foundation for the evolution of electrochemistry and the commercialization of electrochemical energy storage devices, including lithium-ion batteries (LIBs), and aqueous zinc-ion batteries (AZIBs) [1–4]. AZIBs have attracted increasing attention as one of the most promising batteries because of their high theoretical energy capacity (819 mAh g^−1^, 5855 mAh cm^−3^), low electrochemical potential (− 0.76 V *vs.* standard hydrogen electrode), low cost (Zn is the second cheapest metal), and intrinsic non-flammable safety [5]. Furthermore, metallic Zn shows decent stability to oxygen and moisture atmosphere compared with other redox-active metal anodes (e.g., lithium, sodium, and potassium), permitting direct handling in air and a broad range of electrolytes in an aqueous or organic solvent [6]. Despite such inherent advantages of AZIBs over LIBs, the wide adoption and full commercialization of AZIBs is at an immature stage before the persistent issues of existing AZIBs, including dendrite growth, hydrogen evolution reaction (HER), corrosion, and passivation of zinc anode during charging and discharging processes, which leading to short cycling life and low Coulombic efficiency (CE). Inspired by the success of the aforementioned electrolyte development to the commercialization of LIBs [1, 7] and recent success in electrolyte engineering for anode protection [8, 9], we adopt the strategy of the electrolyte development to address these issues and, ultimately, facilitate the robust operation of AZIBs.

**Fig. 1:**
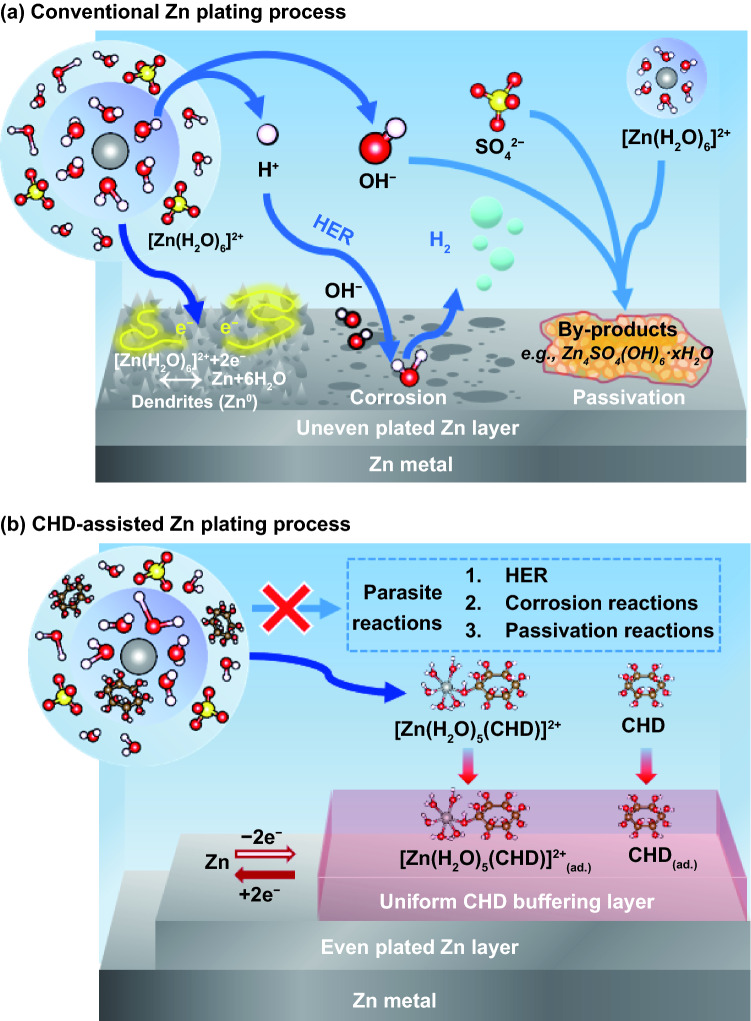
The schematic diagram of zinc (Zn) plating processes in a typical aqueous Zn-ion battery (AZIB). **a** The typical complex ion, [Zn(H_2_O)_6_]^2+^, results in the parasite reactions, including HER, corrosion, dendrite formation, and the passivation on the Zn electrode within ZnSO_4_ aqueous electrolyte; **b** a new complex ion, [Zn(H_2_O)_*m*_(CHD)]^2+^ (optimal *m* = 5), is formed with the presence of CHD in ZnSO_4_ aqueous electrolyte which facilitates the uniform plating/stripping of Zn layer

The electrolyte of AZIBs mainly consists of water and zinc salts (Fig. 1a). It possesses the merits of being non-toxic, non-flammable, environmentally benign, low cost, and high ionic conductivity (~ 0.1 S cm^−1^), in comparison with organic electrolyte that is commonly toxic, flammable, high cost, and low ionic conductivity (~ 1–10 mS cm^−1^). However, the state-of-the-art aqueous electrolyte gives rise to numerous challenges, including significant dendritic Zn growth, severe corrosion, and uneven passivation resulted from parasitic reactions [10]. In ZnSO_4_ aqueous solution, the major form of Zn^2+^ ions is the hydrated Zn^2+^ ions, i.e., [Zn(H_2_O)_6_]^2+^, due to the coordination effect between the Zn^2+^ ion and polarized H_2_O molecules [11]. However, this solvation structure [Zn(H_2_O)_6_]^2+^ could lead to a deprotonation process, producing OH^−^ and H^+^ [12]. On the one hand, the as-generated OH^−^ increase the local pH value and passivate the anode surface by forming insulating precipitate by-products (such as Zn_4_SO_4_(OH)_6_·*x*H_2_O, Zn(OH)_2_, and ZnO) [6, 13, 14]. On the other hand, the as-produced H^+^ ion could induce the corrosion of the Zn anode and hydrogen evolution reaction (HER) on the anode surface.

1 $$2{\text{H}}^{ + }_{{({\text{aq}})}} + {\text{Zn}}_{{({\text{s}})}} \rightleftharpoons {\text{H}}_{{2({\text{gas}})}} + {\text{Zn}}^{2 + }_{{({\text{aq}})}}$$

The product H_2_ could bring in unnecessary risk since increased H_2_ pressure in a sealed battery system would lead to the swelling even explosion of the battery and causes uneven mass transport of the Zn^2+^ ions, resulting in rough Zn plating and low-rate capability.

On the unprotected Zn anode surface, the side reactions could compete against the Zn/Zn^2+^ reversible redox process, leading to the reduced coulombic efficiency and the growth of dendrite [15, 16]. The latter could lead to the penetration of separator and ultimately battery short circuit. It is well established that high activation energy is needed for desolvation of [Zn(H_2_O)_6_]^2+^ to release the Zn^2+^ from the sheath structure. Furthermore, in the course of Zn plating, the energy barrier for Zn nucleation on the Zn electrode surface makes the Zn^2+^ ions prefer to grow on the existing Zn structure instead of nucleation. The growth of isolated micro Zn protrusions results in uneven Zn plating and eventually evolves into pointy Zn dendrite (needle-like), as shown in Fig. 1a.

In order to overcome the above issues of dendrites, HER, corrosion, and passivation, a series of strategies have been proposed: (i) The construction of artificial solid–electrolyte interphase (SEI) on the Zn anode surface, such as ZnS interphase [16], polyvinyl butyral film [15], Zn-based montmorillonite interlayer [17], and polyacrylonitrile coating layer [18], has been proposed to improve the hydrophilicity of the metal electrode, facilitate the rapid transport of Zn^2+^ ions and uniform Zn plating, and prevent the corrosion of the Zn anode. (ii) The alternation of the solvents, such as succinonitrile (SN), is to mediate the solvated Zn^2+^ structure for high-efficient and safe zinc electrodeposition [19]. (iii) The usage of bulky-anion zinc salts, such as Zn(CF_3_SO_3_)_2_ and Zn(TFSI)_2_, leads to the formation of new Zn^2+^ complex ions to replace the problematic [Zn(H_2_O)_6_]^2+^ [20, 21]. (iv) The adoption of a super-concentrated electrolyte such as 20 M LiTFSI is reported by Wang et al. [22]. In this “water-in-salt” electrolyte configuration, salts content is far more than water content. The small amount of water molecules tends to hydrate with the Li^+^, and at the same time, the anions forcefully approach the vicinity of Zn^2+^, leading to the abundant existence of (Zn-TFSI)^+^ rather than [Zn(H_2_O)_6_]^2+^. (v) The addition of low concentration of the functional additives (e.g., glucose [10]) into the pristine electrolyte has been regarded as an affordable, effective, simple, and economical way to enhance the Zn electrode stability [23]. Nevertheless, further exploration of the roles of the electrolyte additives is needed in assuring low-cost, highly effective, and homogenous Zn platting in AZIBs to boost the electrochemical performance and extend the stability of AZIBs.

In this work, we propose the application of a low concentration (0.1 mg mL^−1^) of cyclohexanedodecol (CHD), a polyhydric alcohol, in a diluted ZnSO_4_ aqueous solution (2 M) as shown in Fig. 1b. It is expected that CHD could serve two purposes. Firstly, CHD could be reactive with the hydrated Zn(H_2_O)_6_^2+^ structure and form new hydrated complex ions, mainly [Zn(H_2_O)_*m*_(CHD)]^2+^ to facilitate the rapid desolvation and nucleation in the course of the electrochemical Zn plating. Secondly, CHD could be readily adsorbed on the Zn anode and build a protection layer, which not only facilitates the even and efficient adsorption of [Zn(H_2_O)_*m*_(CHD)]^2+^ and the simultaneously electrochemical plating process, but also prevent the occurrence of the HER reaction and formation of the passivation layer. Experiments and theoretical calculations are carried out to verify the proposed working mechanism of CHD. Due to the efficient functions of CHD electrolyte additive, at the fixed capacity of 1 mAh cm^−2^, a reversible Zn plating/stripping in the Zn|Zn symmetric cells could be achieved up to 2200 h at 2 mA cm^−2^, 1000 h at 5 mA cm^−2^, and 650 h at 10 mA cm^−2^. The Zn|V_2_O_5_ full cells in the CHD-assisted electrolyte retain a high capacity of 175 mAh g^−1^ after 2000 cycles under the high current density of 2 A g^−1^ with the high mass loading of ~ 5 mg cm^−2^ on the cathode.

## Experimental

### Materials Preparation

The Zn foil (purity of 99.99%, thickness of 0.2 mm), copper foil, and stainless steel foil were purchased by Shenzhen Kejing Star Technology. ZnSO_4_·H_2_O powder (purity of ≥ 99.9% trace metals basis), cyclohexanedodecol dehydrate (purity of ≥ 97%), and vanadium (V) oxide (purity of ≥ 98%) were supplied by Sigma-Aldrich Chemical Co.

### Materials Characterization

The morphologies of the electrode surface and elemental analysis were conducted using Hitachi S7100 scanning electron microscopy (SEM) system combined with energy-dispersive X-ray (EDX) spectroscopy. The crystalline analysis of Zn-deposited copper foil was recorded by X-ray diffraction (XRD, Bruker D8 Advance ECO, Germany). Zeta potential was obtained by Particulate Systems (NanoPlus HD). Contact angle was metered on Kruss DSA 258. The in situ optical microscopy was conducted to continuously obtained the visual changes at the Zn anode at the current density of 5 mA cm^−2^ for 20 min. The H-NMR spectra were recorded from a Bruker 400 MHz spectrometer. The nano-scratch was performed by nanoindentation system (Hysitron TI 950) with Berkovich indenter.

### Electrochemical Measurements

Coin cells (CR2032) were assembled for Zn|Zn and SS|SS symmetric cells, Zn|Cu half cells, and Zn|V_2_O_5_ full cells, where Zn is Zn foil, SS is stainless steel foil, Cu is copper foil, and V_2_O_5_ is V_2_O_5_ electrode. The aqueous electrolytes are prepared by deionized water. Unless otherwise indicated, the electrolytes used in this manuscript are 2 M ZnSO_4_ aqueous solution and 0.1 mg mL^−1^ CHD in 2 M ZnSO_4_ aqueous solution, which are abbreviated as ZnSO_4_ electrolyte and ZnSO_4_-CHD electrolyte (or CHD-assisted electrolytes). Specifically, different concentrations (i.e., 0.02, 0.04, 0.1, 0.16, and 0.2 mg mL^−1^) of CHD in 2 M ZnSO_4_ aqueous solution are prepared for the measurement of *zeta* potentials and contact angles. The separator is glass fiber. Electrochemical impedance spectroscopy (EIS) was measured using the electrochemical workstation (CHI660A, Shanghai Chenhua Instrument, *Inc*) on the frequency range between 100 kHz to 10 mHz and amplitude of 5 mV. The linear sweep voltammetry (LSV) was tested in Zn|Cu half cells after standing for 43 h between 0.5 and 1.5 V with the scan rate of 5 mV S^−1^. The Tafel test was conducted in Zn|Zn cells between the − 0.3 to 0.3 V with the scan rate of 1 mV S^−1^. In the Zn|V_2_O_5_ full cells, the 1.0 g V_2_O_5_ powder was treated by 15 mL 2 M NaCl aqueous solutions (2 M) with 72 h stirring [24]. The V_2_O_5_ electrode was fabricated by the mass ratio of 7:2:1 in V_2_O_5_/super-P/PTFE binder. The cycling tests were conducted between the potential of 0.2 and 1.6 V (vs. Zn^2+^/Zn) in LAND-CT2001A battery instrument (Wuhan, China).

### Computational Details

(1) Ab-initio calculations: The spin-polarized DFT calculations were performed using generalized gradient approximation (GGA) parameterized by Perdew–Burke–Ernzerhof (PBE) exchange–correlation functional [25, 26] as implemented in the Vienna Ab Initio Simulation Package (VASP) [27, 28]. The interaction potentials of the core electrons were replaced by projector augmented wave (PAW) [29] pseudopotentials (Zn: 3*d*^10^4*s*^2^, C: 2*s*^2^2*p*^2^, O: 2*s*^2^2*p*^4^, H: 1*s*^1^). The cutoff energy is 600 eV, and a small broadening width of Gaussian smearing (0.05 eV) is used with the *k*-space sampled only by the *Γ* point. The geometry optimization is only performed along the *x* and *y* axes. Along the *z* direction, there is at least 15 Å gap for eliminating the interaction between periodic slabs. The convergence criteria of ionic relaxation and electronic minimization are 0.03 eV Å^−1^ (maximum force on any ion) and 10^–6^ eV, respectively. All these parameters were carefully tested to ensure convergence and accuracy. Semiempirical dispersion correction, namely zero damping DFT-D3 method [30, 31], is used in all the calculations. A 10 × 8 × 2 supercell (320 atoms) with a four-layer Zn slab (001) represents the absorbing surface, and the bottom two layers are kept fixed.

The binding energies (${E}_{b}$) of complex ions are defined as follows:

2 $$E_{b} = E_{{{\text{complex}}}} - E_{{{\text{cation}}}} - E_{m}$$

where $E_{{{\text{complex}}}}$ is the total energy of Zn^2+^ ion complex, $E_{{{\text{cation}}}}$ is the energy of cations, and $E_{m}$ represents the total energy of water or CHD molecules.

The total solvation energies (*E*_total_) of [Zn(H_2_O)_*n*_]^2+^ (*n* = 1 ~ 6) and [Zn(H_2_O)_*n*_(CHD)]^2+^ (*m* = 0 ~ 5) are defined as follows:

3 $$E_{{{\text{total}}}} = E\{ [{\text{Zn}}({\text{H}}_{2} {\text{O}})_{n} ]^{2 + } \} - nE\left( {{\text{H}}_{2} {\text{O}}} \right) - E\left( {{\text{Zn}}^{2 + } } \right)$$

4 $$E_{{{\text{total}}}} = E\left\{ {\left[ {{\text{Zn}}\left( {{\text{H}}_{2} {\text{O}}} \right)_{m} \left( {{\text{CHD}}} \right)} \right]^{2 + } } \right\} - mE\left( {{\text{H}}_{2} {\text{O}}} \right) - E\left( {{\text{Zn}}^{2 + } } \right) - E\left( {{\text{CHD}}} \right)$$

where $E\left\{{\left[\mathrm{Zn}{({\mathrm{H}}_{2}\mathrm{O})}_{n}\right]}^{2+}\right\}$ and $E\left\{{\left[\mathrm{Zn}{\left({\mathrm{H}}_{2}\mathrm{O}\right)}_{m}(\mathrm{CHD})\right]}^{2+}\right\}$ refer to the total energies of solvated ion clusters with the hydration number *n* and *m*. $E\left({\mathrm{H}}_{2}\mathrm{O}\right)$, $E\left({\mathrm{Zn}}^{2+}\right)$, and $E(\mathrm{CHD})$ are the energies of a water molecule, a ${\mathrm{Zn}}^{2+}$ ion, and a CHD molecule, respectively.

During the desolvation process, a solved ion cluster will lose all water molecules of hydration one by one, so the successive desolvation energies ($\Delta E$) can be obtained according to the following equations (*n* = 1 ~ 6, *m* = 0 ~ 5):

5 $$\Delta E=E\left\{{\left[\mathrm{Zn}{\left({\mathrm{H}}_{2}\mathrm{O}\right)}_{6}\right]}^{2+}\right\}-\{nE\left({\mathrm{H}}_{2}\mathrm{O}\right)+E\left\{{\left[\mathrm{Zn}{\left({\mathrm{H}}_{2}\mathrm{O}\right)}_{6-n}\right]}^{2+}\right\}\}$$

6 $$\Delta E=E\left\{{\left[\mathrm{Zn}{\left({\mathrm{H}}_{2}\mathrm{O}\right)}_{5}(\mathrm{CHD})\right]}^{2+}\right\}-\{mE\left({\mathrm{H}}_{2}\mathrm{O}\right)+E\left\{{\left[\mathrm{Zn}{\left({\mathrm{H}}_{2}\mathrm{O}\right)}_{5-m}\left(\mathrm{CHD}\right)\right]}^{2+}\right\}\}$$

The absorbed energies between Zn slab and different molecules are defined as follows:

7 $${E}_{\mathrm{abs}}=E\left({\mathrm{Zn}}_{\mathrm{slab}}+\mathrm{molecules}\right)-E\left({\mathrm{Zn}}_{\mathrm{slab}}\right)-E(\mathrm{molecules})$$

(2) Molecular dynamics simulations: The MD simulation system contains only one Zn^2+^ ion or one Zn^2+^ ion and one CHD molecule surrounded by around 10,000 water molecules. This model is used to investigate the intrinsic hydration behavior of the Zn ion and the CHD molecule. MD simulations were performed in a canonical ensemble using the GROMACS package [32] with Amber force fields (amber14sb) and SPC water model. The size of the box was $10\times 10\times 10$ nm^3^, and periodic boundary conditions were set in all three directions. The electrostatic and van der Waals (vdW) interactions were computed using PME [33] and cutoff methods, respectively. Both methods shared the same cutoff length of 1.0 nm. Two chloride ions (Cl^−^) were added to maintain electric neutrality. H-angles constraining method was used to increase the time step to 2 fs. The temperature was controlled by a V-rescale thermostat [34], and the pressure was controlled by a Berendsen barostat [35]. The system was annealed from 0 to 298 K and maintained at 298 K to reach equilibrium. Then, a 2-ns production simulation was finished for post-processing analysis. The pressure coupling method in the production simulation period was changed to the Parrinello–Rahman method [36].

## Results and Discussion

### Characterizations of the Zinc Deposition Layers

**Fig. 2:**
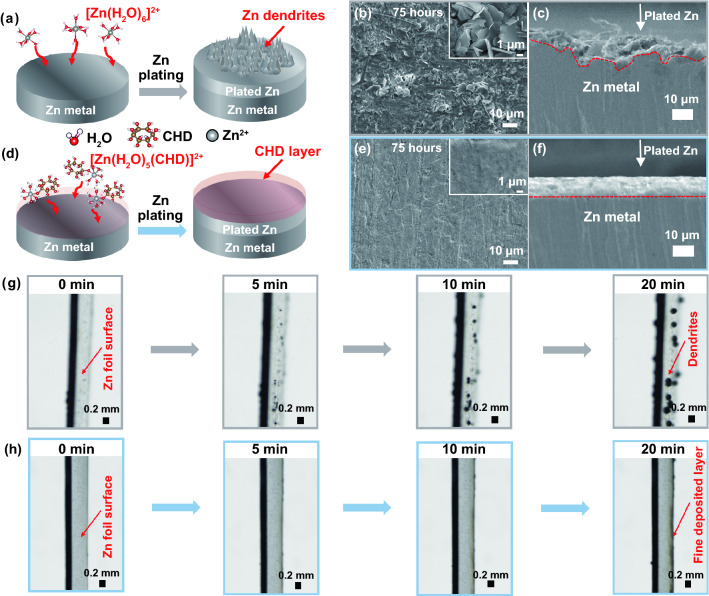
**a–f** Schematic figure of Zn plating process and the corresponding SEM images on Zn foil at 2 mA cm^−2^ and 1 mAh cm^−2^ for 75 h in Zn|Zn cells under ZnSO_4_ (**a–c**) and CHD-assisted ZnSO_4_ electrolytes (**d–f**), respectively. **g–h** In situ optical microscopy images of the Zn deposition process using ZnSO_4_ (**g**) and CHD-assisted ZnSO_4_ electrolyte (**h**)

It is well established that the growth of Zn dendrites during the plating process is mainly due to the parasitic reactions incurred by the [Zn(H_2_O)_6_]^2+^ complex ions in traditional aqueous ZnSO_4_ electrolyte (Fig. 2a–c), while the CHD additive can regulate the solvation behaviors and interfacial properties, resulting in improved plating/stripping performance and suppressed Zn dendrite growth (Fig. 2d–f). To verify this hypothesis, the Zn deposition process was studied via SEM images and in situ optical observation. The SEM images of Zn anode after the electrochemical platting are obtained from top view and side view as shown in Figs. 2b, c, e, f, and S1. This SEM observation was operated after different cycles under the fixed capacity of 1 mAh cm^−2^ and current density of 2 mA cm^−2^. Without the CHD additives, the Zn anode exhibited non-uniform deposition of Zn and side products throughout the surface after 15 cycles (Fig. S1a). This loose and uneven implanting structure cannot isolate the contact between water and electrode, allowing for continuous side reactions between Zn anode and electrolyte, eventually bringing uncontrollable dendritic Zn and by-products after the repeated charge/discharge process as shown in Figs. 2b and S1a-b. Worse still, a rough surface can be observed on the Zn anode after deposition, demonstrating that severe corrosion reactions occurred during the depositing process in the ZnSO_4_ electrolyte (Fig. 2c). In sharp contrast, a [Zn(H_2_O)_*m*_(CHD)]^2+^ solvation structure can form in the presence of CHD additive in ZnSO_4_ electrolyte (Fig. 2d). The generated [Zn(H_2_O)_*m*_(CHD)]^2+^ can facilitate the Zn^2+^ deposition, achieving a flat and stable deposition layer without any surface change after cycles (Figs. 2e and S1c-d) and maintaining a stable plating/stripping process for 1800 cycles (Fig. S1e). The improved stability of Zn anode can be further evidenced by the side view of Zn foil, in which a dense and smooth-plated Zn layer is formed after cycles (Fig. 2f). Moreover, the energy-dispersive spectrometer (EDS) element analysis was conducted to investigate the chemical components of the deposited Zn layer (Fig. S2). Compared with the CHD-assisted ZnSO_4_ electrolyte, the deposited Zn surface in the ZnSO_4_ electrolyte exhibited a higher mass ratio of oxygen and sulfur element while lower content of zinc element, indicating the generation of SO_4_^2−^-based by-products and the loss of zinc resources on Zn electrode.

To eliminate the interference of pristine Zn on the deposited layer, copper (Cu) substrates have been widely applied for Zn electrodeposition because Cu possesses high hydrogen overpotential, high redox potential (0.342 V vs. SHE), and, above all, good affinity (zincophilicity) for Zn [37]. As shown in Fig. S3, the depositing/plating performance on Cu foil was investigated to better understand the positive role of the electrolyte additive CHD in ZnSO_4_ electrolyte. Under the successive deposition of 2 mA cm^−2^ for 1 h, the irregular dendrites and stacked by-products are clearly observed in the bare ZnSO_4_ electrolyte (Fig. S3a-b), resulting in a low CE because of the continuous consumption of the electrolyte during cycling [38]. On the contrary, a homogenous layer without any cracks and dendrites can be seen after Zn deposition (Fig. S3c-d) in the CHD-assisted electrolyte, i.e., [Zn(H_2_O)_*m*_(CHD)]^2+^ electrolyte. As evidenced by the XRD patterns, clear characteristic peaks of zinc metal (JCPDS No. 04-0831) after deposition can be observed in Fig. S4. Specifically, solid and sharp Zn peaks are shown in blank ZnSO_4_ electrolytes due to the aggregation of big Zn particles (dendrites) on the copper substrate. In contrast, small and weak Zn peaks are observed after CHD addition, indicating the presence of low crystalline Zn particles in an ultra-small size [10]. This suggests that the CHD-assisted electrolyte system could reduce the nucleation energy barriers and the deposited Zn particles.

To further illustrate the role of CHD additive, the in situ optical microscopy [8, 10, 39] was executed to observe the in-situ changes at the Zn anode surface in a symmetric Zn|Zn cells under the high-resolution camera (Nikon, SMZ1270) during the galvanostatic deposition and stripping process, as shown in Figs. 2g, h and S5. In the blank ZnSO_4_ aqueous electrolyte (Fig. 2g), numerous Zn nuclei are unevenly distributed on the Zn foil surface after 5 min. After 20 min, the small protrusion gradually grows at the same position and eventually evolves into prominent Zn dendrites. In contrast, after the addition of CHD in the electrolyte (Fig. 2h), a clear Zn foil surface with a uniform and dense Zn plating layer could be monitored in 20 min, indicating that the CHD additives are supportive of impeding the dendrite growth on Zn foil.

The possible H_2_ generation during the Zn plating process could be detected through the in situ electrochemical gas chromatography (EC-GC) [40]. This reactor was a two-neck bottle for gas line transportation and three Zn foil electrodes as the working electrode, counter electrode, and reference electrode. The HER was controlled at the current density of 10 mA cm^−2^ and the Zn plating time of 1 h via the electrochemical station. The H_2_ was characterized by gas chromatography (GC). The quantity of H_2_ released from HER was calculated and normalized as the offset intensity. As shown in Fig. S6, the H_2_ releasing value in the cells with ZnSO_4_ aqueous electrolytes is as high as *ca.* 2.4, while that value is only *ca.* 0.7 in the cells with CHD-assisted electrolytes. The experiments reveal that the HER is significantly suppressed in the CHD-assisted electrolytes. In other words, H_2_O decomposition on the Zn electrode is inhibited under the effect of CHD in aqueous ZnSO_4_ electrolytes.

### Tuning the Solvation Effect of CHD in the ZnSO_4_ Electrolyte

**Fig. 3:**
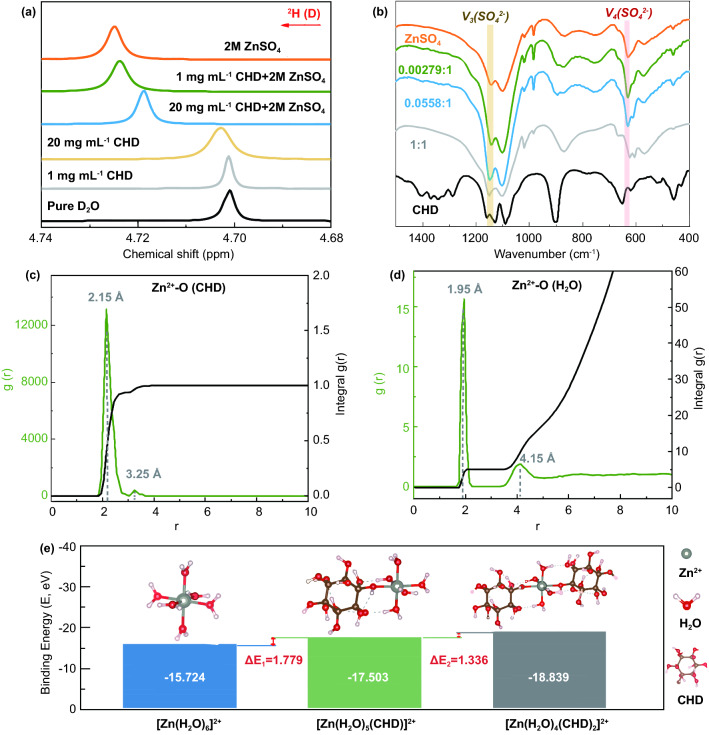
The characterization of the reaction between Zn^2+^ and CHD: **a** H-NMR spectra of H_2_O from pure D_2_O, 1 mg mL^−1^ CHD and 20 mg mL^−1^ CHD, 2 M ZnSO_4_, 1 mg mL^−1^ CHD + 2 M ZnSO_4_, and 10 mg mL^−1^ CHD + 2 M ZnSO_4_ in D_2_O. **b** FTIR spectra of the different mass ratios of CHD/ZnSO_4_ powders. **c, d** The radial distribution functions (RDFs, X—left Y) and the corresponding integral curve (X—right Y) for Zn^2+^-O (CHD) (**c**) and Zn^2+^-O (H_2_O) (**d**) in CHD-assisted Zn-ion aqueous solution. **e** Binding energy comparison of [Zn(H_2_O)_6_]^2+^, [Zn(H_2_O)_5_(CHD)]^2+^, and [Zn(H_2_O)_4_(CHD)_2_]^2+^. The insertions are their corresponding equilibrium structures

Various experimental techniques, including hydrogen nuclear magnetic resonance (H-NMR) and Fourier transform infrared spectroscopy (FTIR), were carried out to investigate the working mechanisms of the CHD-assisted electrolyte structure, i.e., [Zn(H_2_O)_*m*_(CHD)]^2+^ electrolyte. Firstly, the H-NMR at different electrolyte compositions was conducted in D_2_O solvent to monitor the change of coordinated H_2_O molecules. As shown in Fig. 3a, the ^2^H peak is shifted from 4.7009 ppm (pure D_2_O) to 4.7259 ppm (2 M ZnSO_4_) because the surrounding electronic density of water molecules is weakened in the solvation process. Furthermore, after the addition of CHD with concentrations of 1 and 20 mg mL^−1^, the peaks move to 4.7238 and 4.7188 ppm, respectively, indicating that the CHD regulates the Zn^2+^ solvation structure and releases the coordinated water molecules. The 1 mg mL^−1^ CHD and 20 mg mL^−1^ CHD samples without ZnSO_4_ were further tested for better comparison, in which the peaks shift to 4.7012 and 4.7029 ppm, respectively, resulting from the more free waters than that in ZnSO_4_-contained electrolytes. Then, the FTIR was carried out to reveal the mediation of Zn^2+^ coordination environment by the effect of CHD molecules, further confirming the proposed mechanism. Precisely, the mass ratio of CHD/ZnSO_4_ powders of 0.00279/1 and 0.0558/1 corresponds to that in 1 mg mL^−1^ CHD and 20 mg mL^−1^ CHD in the 2 M ZnSO_4_. And two peaks near 1142.82 and 629.55 cm^−1^ are designated to the vibration stretching of *ν*_*3*_(SO_4_^2−^) and *ν*_*4*_(SO_4_^2−^) in ZnSO_4_. When the mass ratio of CHD/ZnSO_4_ is increased from 0.00279/1 to 1/1, an obvious blue shift of *ν*_*3*_(*SO*_*4*_^*2−*^) and a red shift of *ν*_*4*_(SO_4_^2−^) can reach 1151.09 and 623.58 cm^−1^, respectively [41]. The movement of the SO_4_^2−^ peaks in FTIR is ascribed to the CHD molecules changing the interaction between the Zn^2+^ and H_2_O [10]. Overall, the observations of NMR and FTIR demonstrate that CHD could change the Zn^2+^ solvation structure, i.e., from [Zn(H_2_O)_6_]^2+^ electrolyte to [Zn(H_2_O)_*m*_(CHD)]^2+^ electrolyte in aqueous ZnSO_4_ electrolyte.

MD and the DFT calculations were conducted to analyze the roles of CHD in the formation of [Zn(H_2_O)_*m*_(CHD)]^2+^ complex ions in aqueous ZnSO_4_. The MD achieved a good balance in temperature and density, maintaining the high resolution of the calculation (Fig. S7). The radial distribution functions [RDFs, see g(r) axis] of the primary solvation shell (PSS) and average coordination numbers [see integral g(r) axis] of Zn^2+^-O were obtained from the MD, as shown in Figs. 3c, d and S8. In the pure Zn^2+^ ion aqueous solution (Fig. S8), six water molecules coordinate with Zn^2+^ at the PSS, locating from 1.75 to 2.45 Å (0.70 Å in thickness) away from the center metal ion with the maximum value at 1.95 Å. In the proposed [Zn(H_2_O)_*m*_(CHD)]^2+^ electrolyte, as shown in Fig. 3c, the sharp peak of Zn^2+^-O (O from CHD) ranging from 1.85 to 3.95 Å (maximum value at 2.15 Å) originates from an oxygen atom of one hydroxyl functional group from CHD at the PSS, implying one CHD molecule accesses the PSS and replaces one H_2_O molecule. The Zn^2+^ ion could coordinate with the other five water molecules in the range from 1.75 to 2.25 Å (0.50 Å in thickness), with the most probable position at 1.95 Å away from the center (Fig. 3d). Compared with the thickness of PSS in pure Zn^2+^ ion aqueous solution (0.70 Å), CHD-coordinated PSS shrinks to 0.50 Å, implying stronger interaction between Zn^2+^ and water molecules. From this point of view, CHD-coordinated Zn^2+^ complex ion, i.e., [Zn(H_2_O)_*m*_(CHD)]^2+^, is more stable than [Zn(H_2_O)_6_]^2+^. Similar conclusions can be drawn from the binding energies calculated by DFT in Fig. 3e. The coordination of first CHD from [Zn(H_2_O)_6_]^2+^ to Zn(H_2_O)_5_(CHD)]^2+^ can release 1.779 eV per molecule, while the coordination of another CHD from Zn(H_2_O)_5_(CHD)]^2+^ to Zn(H_2_O)_4_(CHD)_2_]^2+^ only can release 1.336 eV per molecule. Considering the extremely low molar ratio of CHD/Zn^2+^ (< 0.0001) and the reduced releasing energy, another CHD tends to coordinate with [Zn(H_2_O)_6_]^2+^ rather than the Zn(H_2_O)_5_(CHD)]^2+^. Thus, it can be inferred that the one-CHD-coordinated Zn^2+^ complex ion (i.e., [Zn(H_2_O)_5_(CHD)]^2+^) is the most energy-preferable (i.e., the most possible) hydrated structure in the electrolyte.

In general, Zn^2+^ solvation structure consists of two shells, i.e., inner and outer shells, as shown in Fig. 1. The H_2_O molecules in the inner shell are relatively stable due to the strong coordination bonds with the center Zn^2+^ ions. In contrast, the H_2_O molecules in the outer shell are relatively free due to the weak long-range electrostatic force. They make up an outer shell of the Zn^2+^ ion coordination sphere and maintain a persistent, albeit relatively weak, attraction with the center Zn^2+^ ion. From Fig. S8, 20.82 H_2_O molecules on average were surrounded at the outer solvation shell from 3.35 to 5.05 Å (1.70 Å in thickness) with the most probable distance of 4.15 Å away from the Zn^2+^ center. As the diameter of the CHD molecule is around 6 Å, its impact will be widened from the PSS to the outer shell, shown in Fig. 3d. The broad peak from 3.35 to 4.85 Å (1.50 Å in thickness) with a maximum value at 4.15 Å is ascribed to the outer hydration layer, corresponding to 16.25 H_2_O molecules on average, further indicating that 4.57 H_2_O molecules on average are excluded by the CHD molecules around the Zn^2+^ center, and similar to the PSS layer, the outer layer also shrinks by 0.2 Å.

In addition to the changes in the coordination environment of center metal cations, CHD molecules also change the flow properties of the electrolyte. Considering one CHD molecule possesses twelve hydroxyl functional groups, which can form a significant number of hydrogen bonds with water molecules, as shown in Fig. S9, the number of hydrogen bonds increases from 10.38 to 17.91 on average per hydrated ion. As a result, CHD-coordinated Zn^2+^ complex ion, namely [Zn(H_2_O)_5_(CHD)]^2+^, hinders the free movement of adjacent water molecules, which means these water molecules are mostly present in a “confined” state. In theory, this can help active water molecules formed during dehydration to settle down, inhibiting the water-splitting reaction or HER.

### Working Mechanism of CHD on Electrode/Electrolyte Interface

**Fig. 4:**
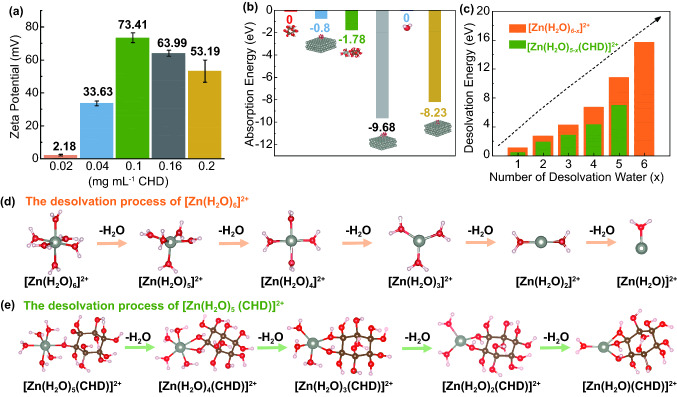
**a** The distribution of the Zeta potentials of Zn^2+^ with different amounts of CHD in ZnSO_4_ aqueous electrolyte with attached error bars. **b** The energy comparison of CHD, [Zn(H_2_O)_5_(CHD)]^2+^, and [Zn(H_2_O)_6_]^2+^ on Zn (001) crystal plane or solution, which is based on the energy zero point of CHD and H_2_O molecules. Insertion is the corresponding absorbed models or dissolved model. The red, blue, green, gray, light blue, and yellow columns and related values are represented as CHD in the solution, CHD on the Zn surface, [Zn(H_2_O)_5_(CHD)]^2+^ in the solution, Zn(H_2_O)_5_(CHD)]^2+^ on the Zn surface, H_2_O in the solution, and [Zn(H_2_O)_6_]^2+^ on the Zn surface, respectively. **c** The desolvation energies of [Zn(H_2_O)_6−*x*_]^2+^ (*x* = 1–6) and [Zn(H_2_O)_5−*x*_(CHD)]^2+^ (*x* = 1–5). **d, e** The molecular geometries of the desolvation processes for [Zn(H_2_O)_6−*x*_]^2+^ (*x* = 1–6) (**d**) and [Zn(H_2_O)_5−*x*_(CHD)]^2+^ (*x* = 1–5) (**e**). The gray, red, white, and brown balls represent zinc, oxygen, hydrogen, and carbon atom, respectively

Since the electrolyte on the electrolyte/Zn anode interface plays a pivotal role in the zinc deposition and parasitic reactions, it is significant to study the effect of CHD molecules toward the Zn crystal on the electrode surface. Experimentally, the affinity of the aqueous solution with the metallic anode could be enhanced after the addition of CHD in the ZnSO_4_ solution, as shown in Fig. S10. The different concentrations of CHD in 2 M ZnSO_4_ aqueous electrolyte present different contact angles, mainly because of their different absorbability and wettability with the Zn anode. Obviously, the 0.1 mg mL^−1^ CHD is more zincophilic and can be homogenously absorbed onto the Zn anode surface [8, 39]. The functions of CHD on the electrolyte/electrode interfaces are characterized by *zeta* potentials measurement (Fig. 4a). A series of CHD concentration in ZnSO_4_ electrolyte (2 M ZnSO_4_ aqueous solution) were prepared as follows: (i) 0.02 mg mL^−1^ CHD; (ii) 0.04 mg mL^−1^ CHD; (iii) CHD; (iv) 0.16 mg mL^−1^ CHD; and (v) 0.2 mg mL^−1^ CHD. The 0.1 mg mL^−1^ CHD in 2 M ZnSO_4_ aqueous electrolyte presents the highest *zeta* potential, approximately 73.41 mV, due to the most stable self-assembled organic layers are formed on the Zn metal surface by CHD molecules and CHD/Zn^2+^ complex [39, 42]. The XPS observed the Zn *2p* and O *1s* on the Zn plates during the different immersion time in 0.1 mg mL^−1^ CHD-modified electrolyte (Fig. S11). It can be found that the energy of Zn surface is changed after its immersion for 24 and 48 h, further illustrating the existence of CHD and CHD/Zn^2+^ complex on Zn surface [43]. The self-assembled layer on the electrode surface was also tested by the nano-scratch techniques (Fig. S12a) [44, 45]. The results show that the organic self-assembled layer in the CHD-assisted ZnSO_4_ electrolyte is more stable mechanically than the inorganic by-products layer in ZnSO_4_ electrolyte. The robust organic protective layer formed by the CHD molecules above the zinc anode surface is important to precisely isolate the electrode surface and the side reactants (e.g., active H_2_O, H^+^, and SO_4_^2−^), while the loose and fragile by-products layers cannot afford a stable battery process, leading to the continuous corrosion and destruction of the metallic anode. Then, the ionic transport capability across the self-assembled layers on the electrode surface was further explored by the exchange current density (*i*_0_) on the Tafel plots analysis (Fig. S12b) [46]. The cells within the CHD-assisted ZnSO_4_ electrolyte presented a higher *i*_0_ of 1.123 mA cm^−2^ than that of 0.802 mA cm^−2^ in ZnSO_4_ electrolyte, suggesting a facile electrochemical kinetic in the regulated organic layers rather than a sluggish ionic transport in the by-products layers. Finally, electrochemical impedance spectroscopy (EIS) was used to characterize the electrochemical behaviors of the pre-formed buffering layer in electrolytes during the cycling (Fig. S12c) [8]. Before the cycle, the charge transfer resistance (*R*_ct_) value in the cells using CHD-assisted ZnSO_4_ electrolyte was higher than that value in ZnSO_4_ electrolyte, which is caused by the adsorption of a large amount of [Zn(H_2_O)_5_(CHD)]^2+^ on the Zn anode. After the cycles, the *R*_ct_ value significantly decreases in the cell containing CHD, indicating an enhanced ionic conductivity on the Zn anode surface during the battery process, while, in the Zn|Zn cells without CHD additives, the *R*_ct_ value increases dramatically after the first cycle due to the massive dead Zn dendrites and heavily aggregated by-products on Zn foil. According to the above results, it can be obtained that the generation of a robust organic adsorption layer in CHD-containing electrolyte could effectively smooth the surface texture of Zn anode and provide excellent protection to the Zn foil to suppress the side reactions.

Theoretically, the DFT methods were performed to calculate the absorption ability between the different small molecules and Zn (001) crystal plane. As illustrated in Fig. 4b, the adsorption energy of [Zn(H_2_O)_5_(CHD)]^2+^ (− 9.68 eV, gray column) was higher than that of CHD (− 0.8 eV, blue column) and [Zn(H_2_O)_6_]^2+^ (− 8.23 eV, yellow column) on the Zn surface, also presenting higher energy than [Zn(H_2_O)_5_(CHD)]^2+^ dissolved in the solution (− 1.78 eV, green column. All these structures are calculated based on the original structures of CHD and H_2_O. This result indicates the strong adsorption between the [Zn(H_2_O)_5_(CHD)]^2+^ and the Zn surface. This matches with the experimental results well. Considering the confining effect of CHD-coordinated Zn^2+^ complex ion on nearby water molecules, it can infer that the [Zn(H_2_O)_5_(CHD)]^2+^ prefers to anchor on the Zn surface to suppress the uncontrollable Zn dendrites and water-induced side reactions (i.e., HER, corrosion, and passivation reaction). Thus, free H_2_O molecules can be strongly ruled out from the Zn anode surface by the uniform [Zn(H_2_O)_5_(CHD)]^2+^ buffering layer.

The desolvation energy is a significant criterion to estimate the quality of coating, which has been illustrated by the DFT calculation in Fig. 4c–e. The stepwise desolvation energies in [Zn(H_2_O)_5−*x*_(CHD)]^2+^ (*x* = 1–5) are between 0.442 to 7.003 eV, which are much lower than that in [Zn(H_2_O)_6−*x*_]^2+^ (*x* = 1–6). This should mainly attribute to the multiple hydroxyl functional groups, which can coordinate with the center Zn^2+^ ion effectively and significantly reduce the energy barriers of the desolvation process (Fig. 4e). Thus, the [Zn(H_2_O)_5−*x*_(CHD)]^2+^ (*x* = 1–5) cluster can easily de-solvate the coordinating water molecules in the modified Zn^2+^ solvation structure, facilitating the stable and smooth Zn plating/stripping to improve the rate capability and long cycling life.

### Electrochemical Characterization of CHD Effect in Zn Deposition/Stripping Process

The ionic conductivity and electrochemical stability are two critical factors to evaluate the quality of the electrolyte. The EIS based on stainless steel cell is used to probe the ionic conductivity of the electrolyte with and without CHD additives. The Zn^2+^ ionic conductivity in the electrolyte (*σ*_Zn_^2+^) could be derived according to the equation of *σ*_Zn_^2+^ = *L*/(*R*_*b*_ × *S*), where *L* is the thickness of the separator, *S* is the contact area between the stainless steel foil and the separators, and *R*_*b*_ is the intercept of the Nyquist curves in the high frequency and defied as the bulk resistance [47]. The *R*_*b*_ value is significant to evaluate the ionic conductivity of the different electrolytes within the same battery test conditions. As shown in Fig. S13a, in contrast to the ZnSO_4_ electrolyte, the CHD-assisted ZnSO_4_ electrolyte shows a lower *R*_*b*_ value, presenting a higher *σ*_Zn_^2+^ value to facilitate a fast transport of Zn ions in the electrolyte. Linear sweep voltammetry (LSV) is a vital measurement to identify the oxidation potential of the electrolyte [48]. As shown in Fig. S13b, the decomposition potential of the CHD-assisted ZnSO_4_ electrolyte is higher than that in ZnSO_4_ electrolyte, exhibiting that the addition of CHD is beneficial to improve the electrochemical stability of the aqueous electrolyte. In a word, the presence of CHD plays a positive role in increasing the ionic diffusion rate and extending the available voltage windows of the electrolyte.

**Fig. 5:**
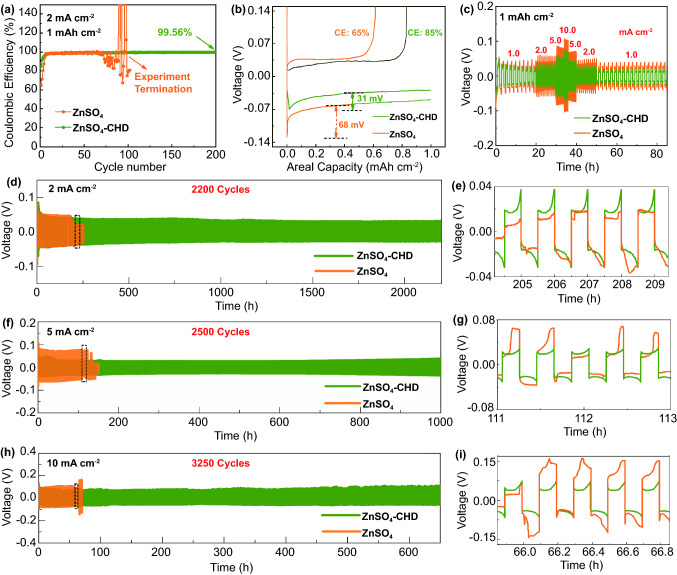
**a** The coulombic efficiency of the Zn plating/stripping at 1 mA cm^−2^ with the fixed capacity of 1 mAh cm^−2^ in Zn|Cu cells; **b** voltage profiles of Zn plating/stripping process on the copper mesh; **c** Rate galvanostatic cycling of Zn|Zn symmetrical cell at 1, 2, 5, and 10 mA cm^−2^ with the capacity of 1 mAh cm^−2^; **d–i** long-term galvanostatic cycling of Zn|Zn symmetrical cell at 2 mA cm^−2^ (**d–e**), 5 mA cm^−2^ (**f–g**) and 10 mA cm^−2^ (**h–i**) with the fixed capacity of 1 mAh cm^−2^. **e**, **g**, **i** are the magnified curves of the selected time (i.e., the dotted rectangles) in (**d**, **f**, **h**), respectively

The CE was obtained to reveal the reversibility and efficiency of the battery process. The Zn|Cu cells in CHD-assisted ZnSO_4_ electrolyte and ZnSO_4_ electrolyte were prepared to characterize the Zn plating/stripping on the copper substrate with plating for 30 min and stripping until 1.0 V at the 2 mA cm^−2^ as presented in Fig. 5a. The CE in the ZnSO_4_ electrolyte dramatically fluctuates and quickly decays after around 60 cycles, reflecting the irregular Zn deposition on the copper mesh. On the contrary, a high CE of 99.56% and stable cyclability could be maintained after 200 cycles with the CHD additives in the ZnSO_4_ aqueous solution, attributing to the fact that the CHD effectively regulates Zn plating and stripping process. More details about Zn plating and stripping behaviors were revealed by initial capacity-voltage profiles, as illustrated in Fig. 5b. The pristine ZnSO_4_ electrolyte exhibits a larger charge/discharge polarization compared with the CHD-assisted one, which can be evidenced by the wider voltage gap of 99 mV in ZnSO_4_ electrolyte than 69 mV in CHD-assisted ZnSO_4_ electrolyte. The high overpotential could result in increased polarization, poor CE, damage of the electrode, and even battery failure. The nucleation overpotential of CHD-assisted ZnSO_4_ electrolyte (31 mV) is much lower than that in bare ZnSO_4_ electrolyte (68 mV), indicating that the CHD-assembled organic layers can activate the zinc nucleation process and reduce the resistance of zinc plating in the first cycles. The reduction of zinc nucleation energy barriers promotes the generation of small zinc nucleus on electrode rather than the fast growth of disordered Zn dendrites [49]. This result corresponds with the SEM observations in Fig. 2 and XRD patterns in Fig. S4.

In order to further demonstrate the cycling stability and high rate capability of the modified electrolyte containing CHD additives, the Zn|Zn symmetric cells by galvanostatic measurements at different current densities were analyzed as summarized in Fig. 5c–i. The stability of the Zn anode was firstly tested at the stepped current density of 1, 2, 5, and 10 mA cm^−2^ with 1 mAh cm^−2^ for Zn plating and stripping. The higher overpotential at each rate stage was found on the cells without CHD additives. However, the cells in CHD-assisted ZnSO_4_ electrolyte exhibit a gradually reduced overpotential and greatly stable voltage hysteresis after 40 cycles. It suggests that the CHD additives accelerate the ion mobility rate during the Zn deposition/stripping process, which is correspondence with the lower *R*_ct_ after cycle in Fig. S12c. EIS and chronoamperometry experiments were performed with the constant potential of 10 mV for 1000 s in Zn|Zn symmetric cells [50]. The calculated Zn^2+^ transfer numbers in ZnSO_4_ aqueous and ZnSO_4_-CHD aqueous electrolytes are 0.45 and 0.72, respectively. This again demonstrates that the CHD additive could accelerate the overall Zn^2+^ ion mobility rate during the Zn deposition/stripping processes.

Then, the long-term cycling test in Zn|Zn cells was conducted using the electrolyte with and without CHD additives. It can be found that, with a fixed area capacity of 1 mAh cm^−2^, cells with CHD additives are stable cycling up to 2200 h at 2.0 mA cm^−2^, 1000 h at 5.0 mA cm^−2^, and 650 h at 10.0 mA cm^−2^, respectively. This ultra-long lifespan of the CHD-assisted electrolyte is superior to many previous reports (Table S1). The low polarization and stable voltage profiles in the cells containing CHD are beneficial to guild the Zn nucleus energy barriers to achieving the dendrite-free Zinc anode in the even plating process. In contrast, under the same testing parameters, the fluctuant and increased polarization in voltage, followed by a sudden drop-off at the cycling process, appeared in cells without CHD additives. This phenomenon can be clearly observed in the enlarged voltage profiles (Fig. 5e, g, i). The unstable voltage profiles in cells using ZnSO_4_ electrolyte after several cycles are attributed to the severe parasitic reactions on the zinc anode, where uniform, non-ionic conducting by-products layers and H_2_ gas are generated.

### Electrochemical Characterization of Zn|V_2_O_5_ Full Cells

**Fig. 6:**
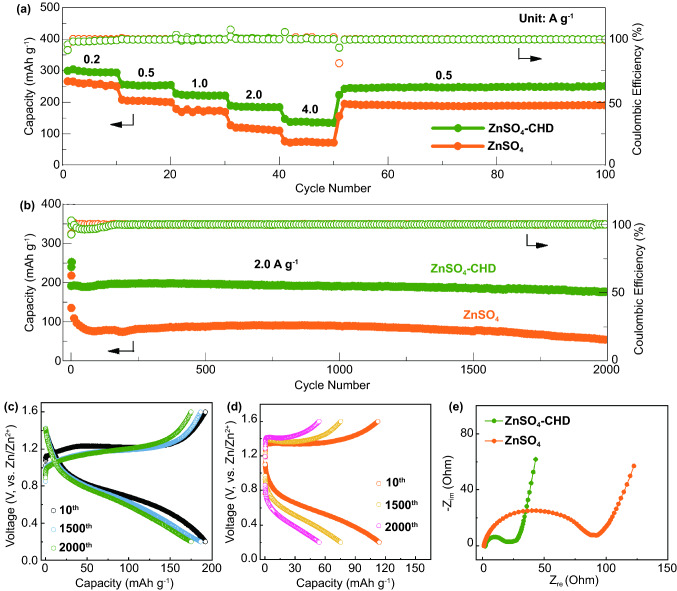
The Zn|V_2_O_5_ full cells performance with and without the CHD additives: **a** Rate performance from 0.2 to 4.0 A g^−1^. **b** Long-term cycling performance at 2.0 A g^−1^ after the activation at 0.2 A g^−1^ for three cycles. **c, d** Voltage profiles at 2.0 A g^−1^ for the 10th, 1500^th^, and 2000th cycle with (**c**) and without (**d**) CHD additives. **e** EIS test with and without CHD additives after the 10th cycle at 200 mA g^−1^

The vanadium oxide (V_2_O_5_) has attracted great interest as promising cathode material for high energy density and high rate capability of AZIBs [24]. However, the V_2_O_5_ cathode presents significant deteriorative electrochemical performance with the increase of electrode mass loading because of the sluggish ionic conductivities and the water-induced parasitic reactions [51]. At the same time, the low mass loading limits the practical tap density and energy density. Herein, we successfully performed a high mass loading (~ 5 mg cm^−2^) V_2_O_5_ cathode in rechargeable AZIBs using CHD electrolyte additives because of the enhanced ionic conductivity and protective effect in the CHD-modified electrolyte. The CHD-assisted electrolyte delivers excellent and competitive performance in Zn|V_2_O_5_ full cells (Table S2). The electrochemical tests were conducted at the voltage range of 0.2–1.6 V (vs. Zn/Zn^2+^). As shown in Fig. 6a, the Zn|V_2_O_5_ full cells using CHD-assisted electrolyte deliver average specific capacities of 300, 252, 231, 189, and 141 mAh g^−1^ at the charge/discharge rate of 0.2, 0.5, 1.0, 2.0, and 4.0 A g^−1^, respectively. Furthermore, a specific capacity of 245 mAh g^−1^ can remain after the current density comes back to 0.5 A g^−1^, outperforming the rate capability of the cells using ZnSO_4_ electrolyte in which there is a slight capacity delay at the low current density of 0.2 A g^−1^ and significantly decreased specific capacity at high current densities (e.g., 74 mAh g^−1^ at 4.0 A g^−1^). Moreover, as shown in Fig. S14, the charge–discharge curves show stable potential plateaus at different current densities in CHD-assisted electrolyte (Fig. S14a), whereas unstable voltage platforms with a high potential gap of 0.9 V can be observed at 4.0 A g^−1^ in pure ZnSO_4_ electrolyte (Fig. S14b). Long-term cycling was further conducted to study the battery performance in Zn|V_2_O_5_ full cells. As shown in Fig. 6b, the cells using ZnSO_4_ electrolyte deliver a rapid capacity decay in the initial 80 cycles and can keep a low specific capacity of only 54 mAh g^−1^ after 2000 cycles with a fast capacity decay of 0.03% per cycle. However, with the CHD additive, the specific capacity of Zn|V_2_O_5_ cells reaches up to 175 mAh g^−1^ after 2000 cycles with a low capacity decay of 0.004% per cycle. Meanwhile, when comparing the voltage platforms during cycling, the full cells using CHD additives maintain a stable voltage plateau after 2000 cycles (Fig. 6c) rather than the unstable charge–discharge platforms and increased voltage gaps in ZnSO_4_ electrolyte (Fig. 6d). Finally, the EIS test was carried out on Zn|V_2_O_5_ cells after 10th cycles at 200 mA g^−1^ (Fig. 6e). The Nyquist curves of Zn|V_2_O_5_ cells in both electrolytes exhibit a semicircle for *R*_ct_ values in the high-frequency region and a line for the diffusion resistance in the low-frequency region. The cells using ZnSO_4_ electrolytes show a large *R*_ct_ value because of the deposition of dead Zn and side products on the electrode. Conversely, the full cells in CHD-assisted electrolytes have a much smaller *R*_ct_ value, corresponding to the improved Zn^2+^ transport and the prevention of side reactions on the electrode. The excellent battery performance in the full cells further demonstrates the significance of CHD additive in achieving high reversible capacities and long cycling life for AZIBs.

## Conclusion

In summary, the CHD, a small organic molecule with polyhydric groups, is applied into the typical and cheap ZnSO_4_ aqueous electrolyte to effectively stabilize the Zn anode in the AZIBs. The solvation shells of Zn^2+^ cations in the electrolyte and surface coverage situation on the Zn electrode are simultaneously restructured after the addition of CHD molecules, which have been verified by materials characterizations and MD associated with the DFT theoretical simulations. The reduced desolvation energy and nucleation barriers in the CHD-assisted ZnSO_4_ electrolyte help smooth Zn deposition without the dead Zn particles. The adsorption of [Zn(H_2_O)_5_(CHD)]^2+^ complexes on the electrode surface effectively decreases the amount of free H_2_O near the Zn anode due to their strong hydrogen bond effect toward the water molecules. The electrochemical test results show long-term cycling stability of Zn anode in the Zn|Zn cells up to 650 h at a high current density of 10.0 mA cm^−2^ under the plating capacity of 1 mAh cm^−2^. The Zn|V_2_O_5_ full cells using CHD additives can achieve a high retained capacity of 175 mAh g^−1^ after 2000 cycles with the high capacity retention of 92% under the high mass loading of ~ 5 mg cm^−2^. Such remarkable long cycling lifespan and capacity performance for the Zn|V_2_O_5_ full cells under the practical operation conditions make a breakthrough in AZIBs, demonstrating the significant impact of CHD additives in improving the quality of AZIBs.

## Supplementary Information

Below is the link to the electronic supplementary material.

Supplementary file1 (PDF 1572 kb)
