# Supplementary material for: Cyclohexanedodecol-Assisted Interfacial Engineering for Robust and High-Performance Zinc Metal Anode
Source: Nanomicro Lett. 2022 Apr 19;14:110. doi: 10.1007/s40820-022-00846-0 (PMC9019003; doi:10.1007/s40820-022-00846-0)
Supplement: Supplementary file 1 — Supplementary file1 (PDF 1572 kb) [file 40820_2022_846_MOESM1_ESM.pdf]

Supporting Information for

Cyclohexanedodecol-Assisted Interfacial Engineering for Robust and High-Performance Zinc Metal Anode

Zhenzhen Wu[1,†], Meng Li[1,†], Yuhui Tian[1], Hao Chen[1], Shao-Jian Zhang[2], Chuang Sun[3], Chengpeng Li[4], Milton Kiefel[4], Chao Lai[3], Zhan Lin[2,]*, Shanqing Zhang[1,]*

[1]Centre for Clean Environment and Energy, Griffith University, Gold Coast 4222, Australia

[2]Guangzhou Key Laboratory of Clean Transportation Energy Chemistry, School of Chemical Engineering and Light Industry, Guangdong University of Technology, Guangzhou 510006, P. R. China

[3]School of Chemistry and Materials Science, Jiangsu Normal University, Xuzhou 221116, P. R. China

[4]Institute for Glycomics, Griffith University, Gold Coast 4222, Australia

†Zhenzhen Wu and Meng Li contributed equally to this work.

*Correspondence authors. E-mail: zhanlin@gdut.edu.cn (Z. Lin), s.zhang@griffith.edu.au (S.Q. Zhang)

Supplementary Figures and Tables

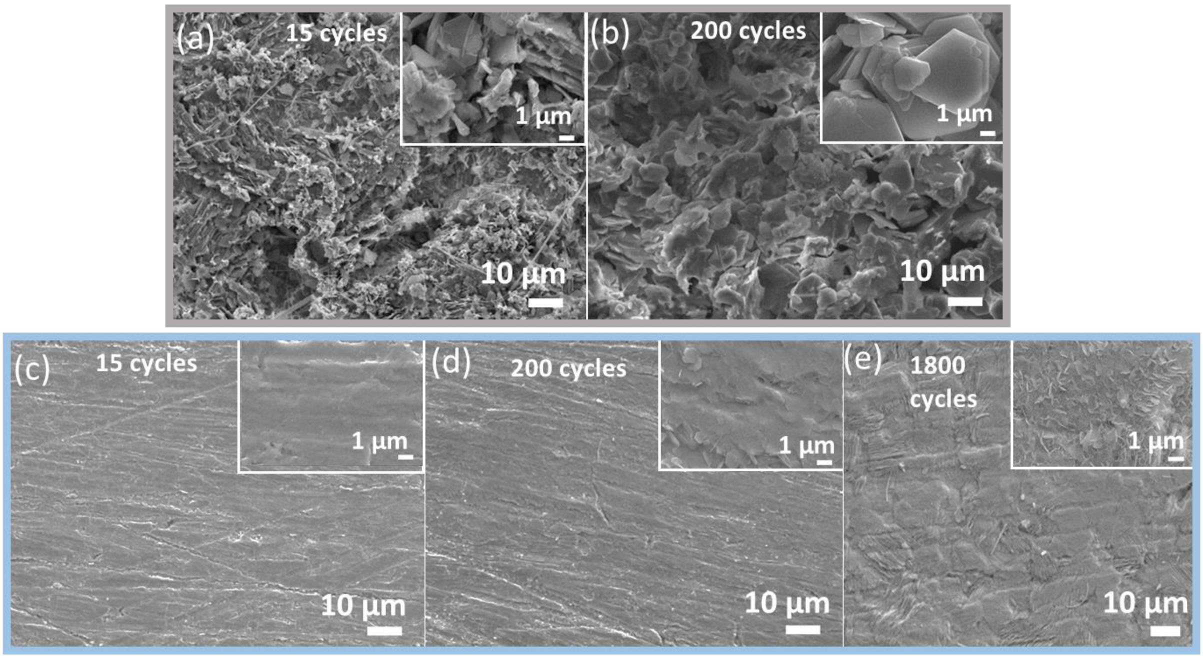

Fig. S1 The morphologies and of Zn anode after cycling at a capacity of 1 mAh cm^{-2} and current density of 2 mA cm^{-2} in $ZnSO_4$ electrolyte (**a-b**) and $ZnSO_4$-CHD electrolyte (**c-e**): **a** and **c** are after 15 cycles, **b** and **d** are after 200 h, **e** is after 1800 h. The bar is 10 µm. Insertion is the magnified image with a scale bar of 1 µm

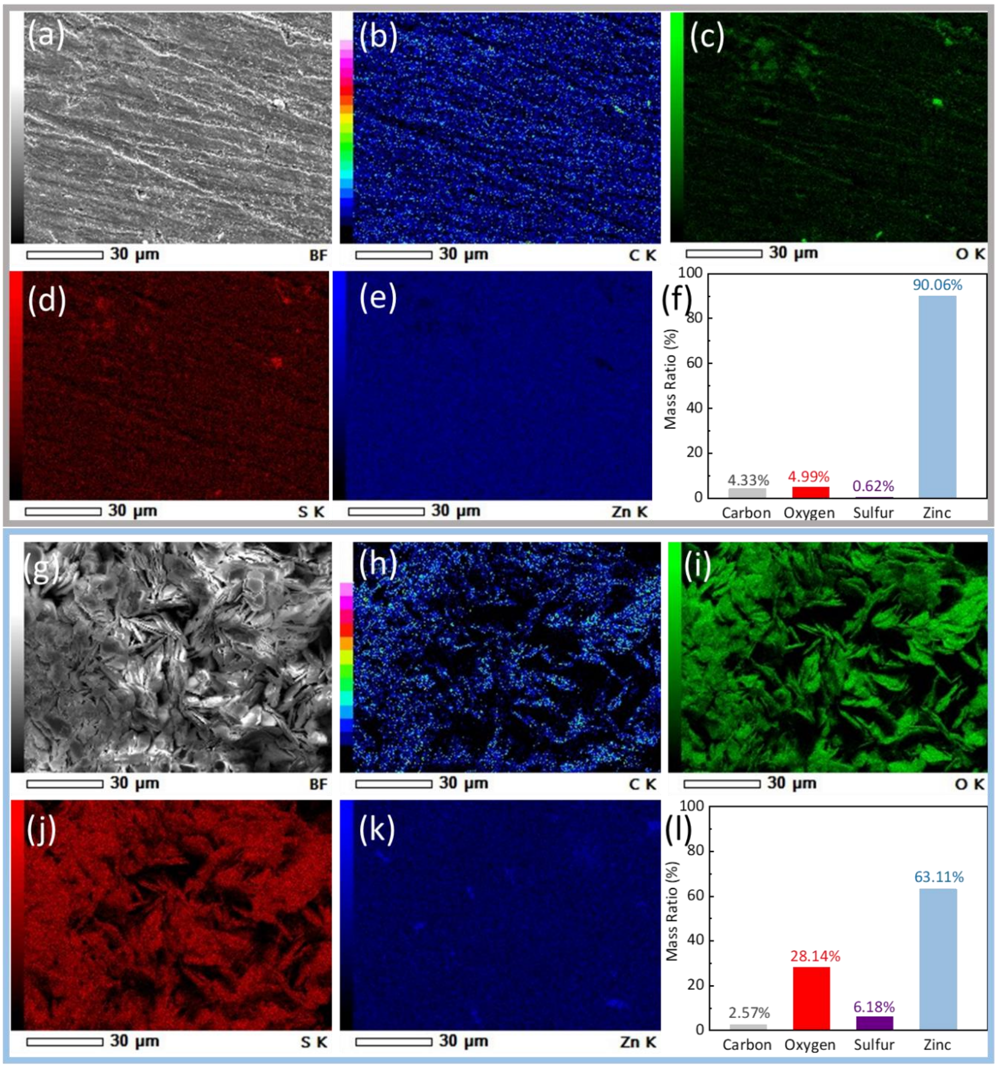

Fig. S2 Morphology and element analysis of metal Zn anode after 200 cycles at 1 mAh cm^{-2} and 2 mA cm^{-2}. SEM images of Zn anode in $ZnSO_4$-CHD electrolyte (**a**) and in $ZnSO_4$ electrolyte (**c**); Elements content of Zn surface by SEM-EDS method in $ZnSO_4$-CHD electrolyte (**b**) and in $ZnSO_4$ electrolyte (**d**)

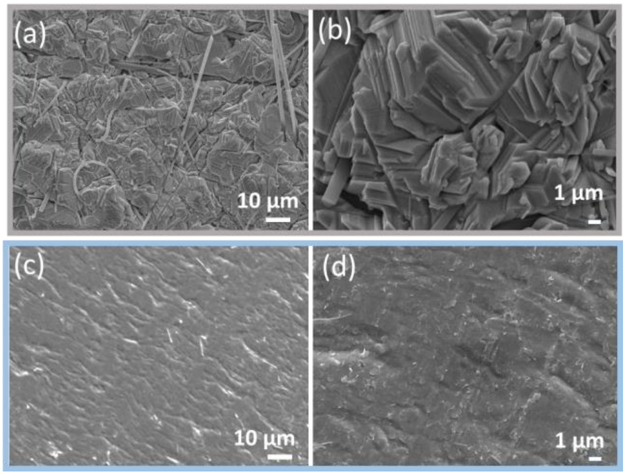

Fig. S3 Top-view SEM images of Zn deposition on Cu foil at 2 mA cm^{-2} for 1 h without (**a-b**) and with CHD additives (**c-d**) in $ZnSO_4$ aqueous solution.

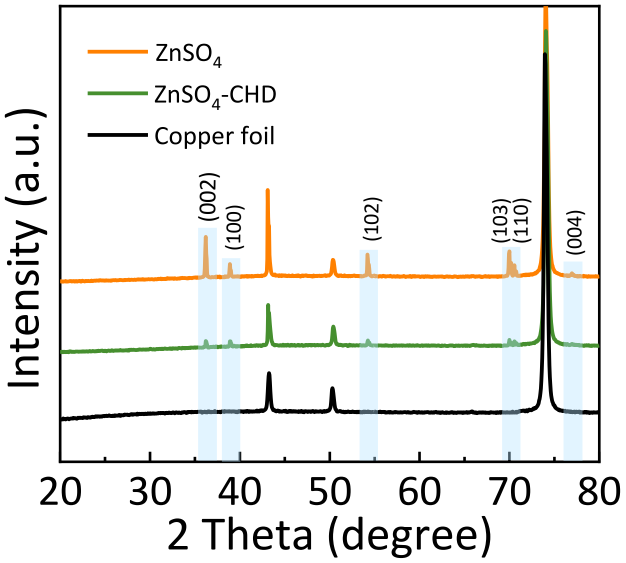

Fig. S4 XRD patterns of bare copper foil and that after the Zn deposition for 2 hours at 1mA cm^{-2}, 2mAh cm^{-2} with and without CHD additives

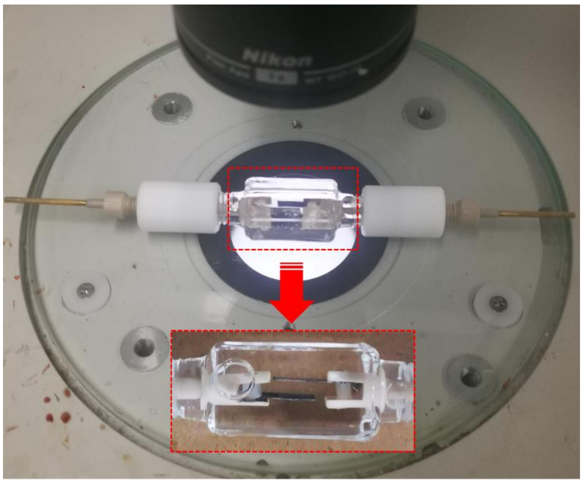

Fig. S5 The *in-situ* optical microscopy system to observe the Zn anode surface during the constant Zn plating process

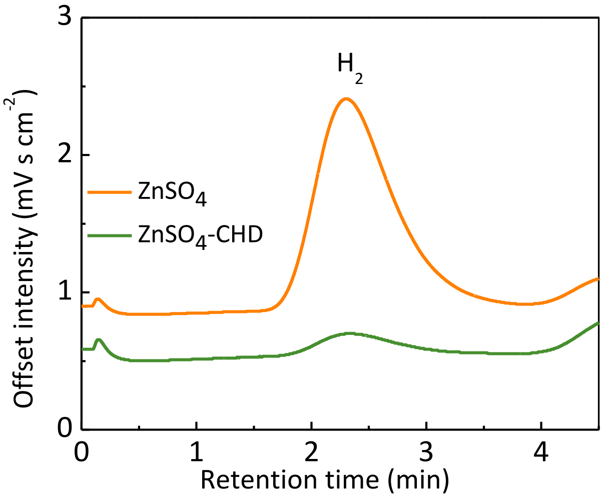

Fig. S6 *In-situ* EC-GC profiles during plating for 1h at the current density of 10 mA cm^{-2}

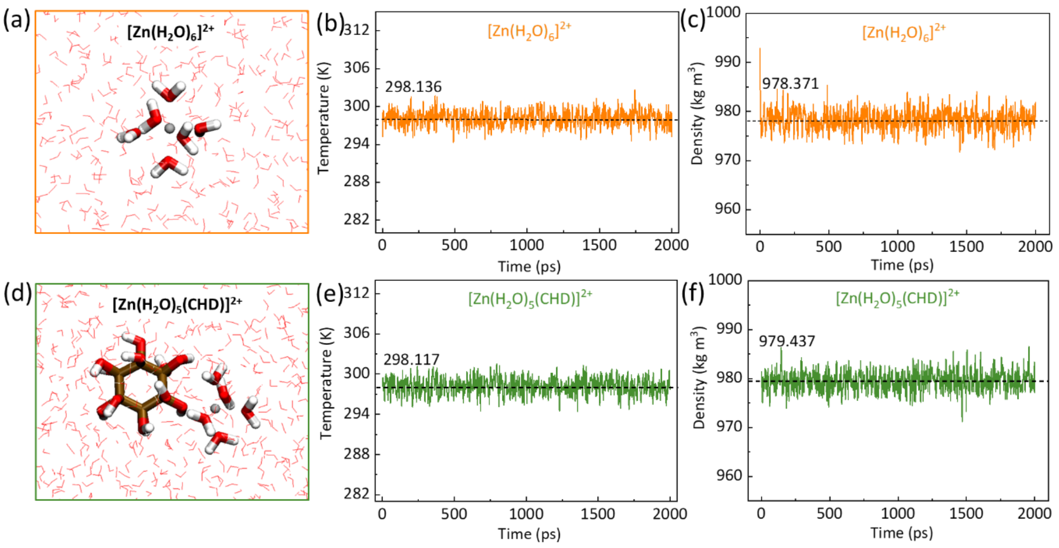

Fig. S7 The MD simulations at 298.15K and 2000ps of $ZnSO_4$ system electrolyte (**a-c**) and $ZnSO_4$-CHD system electrolyte (**d-f**): The snapshot of representative Zn-solvation sheath in the $ZnSO_4$ system (**a**) and $ZnSO_4$-CHD system (**d**), the red, white, grey and blue sticks represent oxygen, hydrogen, zinc, and carbon atoms, respectively; The time versus temperature figure in the $ZnSO_4$ system (**b**) and $ZnSO_4$-CHD system (**e**); The time versus density figure in the $ZnSO_4$ system (**c**) and $ZnSO_4$-CHD system (**f**)

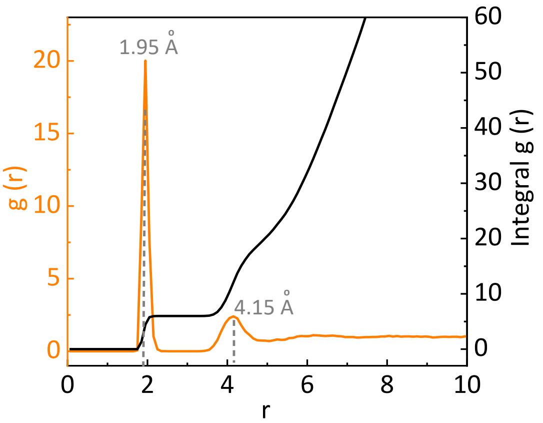

Fig. S8 The MD simulations at 298.15K and 100ps of Zn^{2+}-O (H_2O) in $ZnSO_4$ system electrolyte

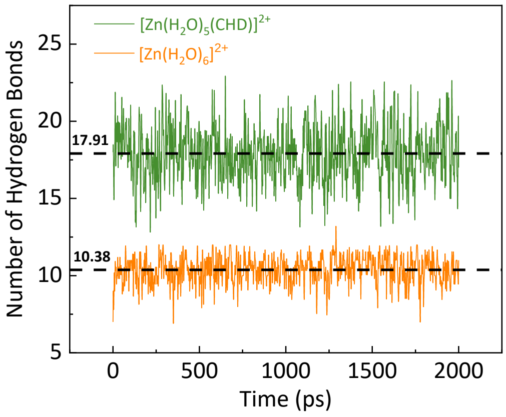

Fig. S9 The number of hydrogen bonds around the molecular cluster of $[Zn(H_2O)_5(CHD)]^{2+}$ and $[Zn(H_2O)_6]^{2+}$ in the electrolyte from the MD simulation

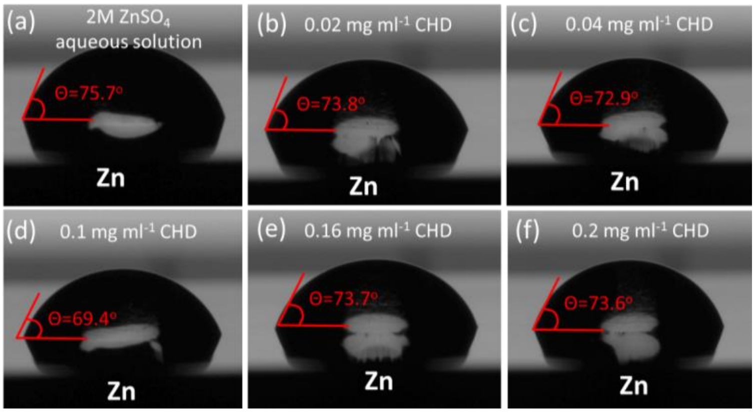

Fig. S10 Measurements of contact angles after the droplet stable for 3 minutes. Contact angles of electrolytes with blank electrolyte (**a**), 0.02mg ml^{-1} (**b**), 0.04mg ml^{-1} (**c**), 0.1mg ml^{-1} (**d**), 0.16mg ml^{-1} (**e**), and 0.2 mg ml^{-1} (**f**) CHD additive on Zinc electrode surface

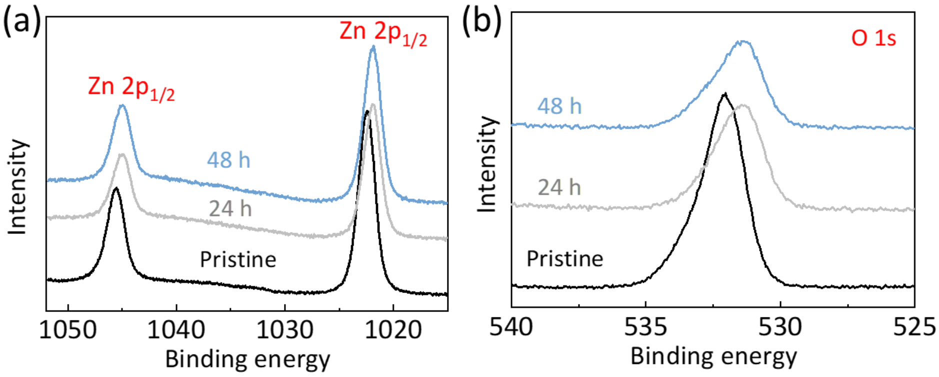

Fig. S11 The XPS spectra of Zn foil in the pristine state and immersion in CHD-assisted electrolyte for 24 and 48 hours. (**a**) *Zn 2p*; (**b**) *O 1s*

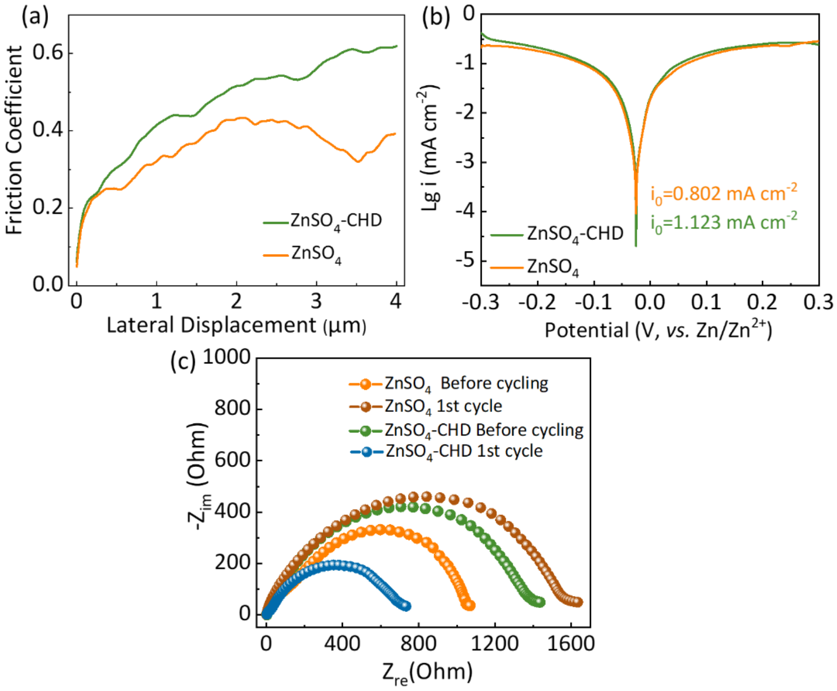

Fig. S12 (**a**) Nanoscratch test of copper foil surface in $ZnSO_4$-CHD and $ZnSO_4$ aqueous solution. (**b**) Tafel curves of Zn/Zn symmetric cells. (**c**) EIS curves of Zn|Zn symmetric cells before and after 1st cycle. The battery was tested at 1mAh cm^{-2} and 2mA cm^{-2}

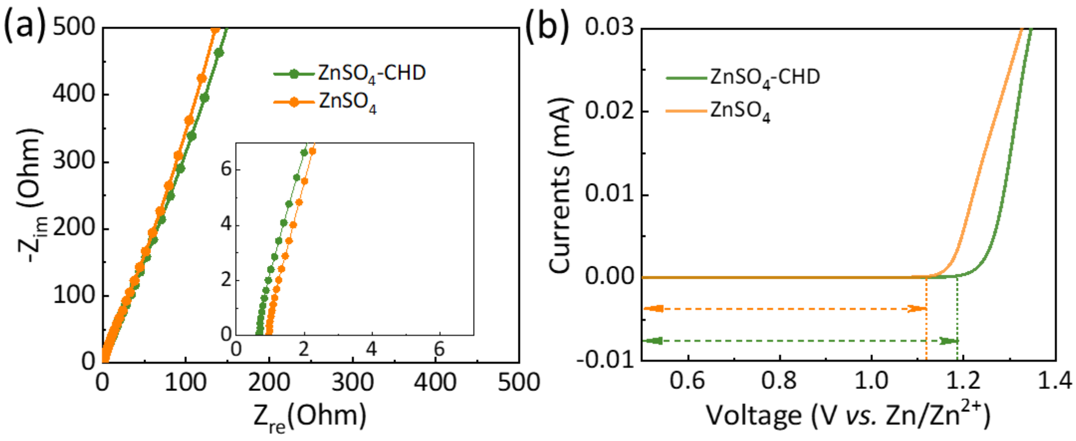

Fig. S13 (**a**) EIS of stain steel-stain steel cell in the electrolyte with and without CHD additives. Insertion is the magnified curves of EIS at the high-frequency region. (**b**) linear sweep voltammetry (LSV) with and without CHD additives in Zn|Cu cells

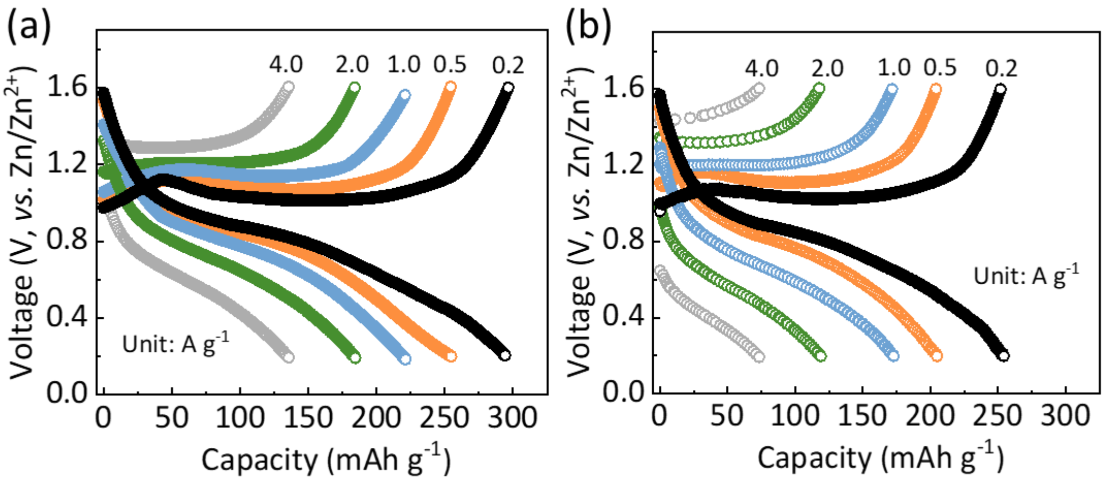

Fig. S14 (**a-b**) Charge-discharge profiles of Zn|V_2O_5 full cell from 0.2 to 4 A g^{-1} with (**a**) and without (**b**) CHD additives

Table S1 Comparison of cycling performance for the modified electrolytes with various additives in Zn|Zn symmetric cells

The modified electrolytes	Lifespan	Refs.
**CHD + 2 M $ZnSO_4$ in H_2O**	2 mA cm^{-2}, 1 mAh cm^{-2} for 2200 h<br>5 mA cm^{-2}, 1 mAh cm^{-2} for 1000 h<br>10 mA cm^{-2}, 1 mAh cm^{-2} for 650 h	This work
**1 M Zn(TFSI) + 20 M LiTFSI in H_2O**	0.2 mA cm^{-2}, 0.035 mAh cm^{-2} for 170 h	[S1]
**3 M $Zn(CF_3SO_3)_2$ in H_2O**	0.1 mA cm^{-2}, 0.1mAh cm^{-2} for 800 h	[S2]
**Ti_3C_2TX MXene + 2 M $ZnSO_4$ in H_2O**	1 mA cm^{-2}, 1 mAh cm^{-2} for 500 h	[S3]
**Glucose + 1 M $ZnSO_4$ in H_2O**	1 mA cm^{-2}, 1 mAh cm^{-2} for 2000 h	[S4]
**DMSO + 1.6 M $ZnCl_2$ in H_2O**	0.5 mA cm^{-2}, 0.5 mAh cm^{-2} for 1000 h	[S5]
**PAM + 1 M $ZnSO_4$ + 0.5 M Na_2SO_4 in H_2O**	1 mA cm^{-2}, 1 mAh cm^{-2} for 180 h	[S6]
**Diethyl ether + 3M $Zn(CF_3SO_3)_2$ in H_2O**	0.2 mA cm^{-2}, 0.2 mAh cm^{-2} for 250 h	[S7]
**$Zn(ClO_4)_2$*$6H_2O$ in SN**	0.05 mA cm^{-2}, 0.5 mAh cm^{-2} for 800 h	[S8]
**0.5 M ZnTFMS in DMF**	1 mA cm^{-2}, 1 mAh cm^{-2} for 2800 h	[S9]

Table S2 Comparison of the electrochemical performance in Zn|V_2O_5 full cells

V_2O_5 mass loading	Electrolyte	Specific capacity	Cycling stability	Refs.
***ca.* 5.0 mg cm^{-2}**	CHD + 2 M $ZnSO_4$ in H_2O	300 mAh g^{-1} (200 mA g^{-1})	2000 cycles (2 A g^{-1})	This work
**2.5 mg cm^{-2}**	3 M $Zn(CF_3SO_3)_2$ in H_2O	381 mAh g^{-1} (60 mA g^{-1})	950 cycles (6 A g^{-1})	[S10]
**N/A**	3 M $ZnSO_4$ in H_2O	224 mAh g^{-1} (100 mA g^{-1})	400 cycles (2 A g^{-1})	[S11]
**N/A**	21 MLiTFSI + 1 M $Zn(CF_3SO_3)_2$ in H_2O	238 mAh g^{-1} (50 mA g^{-1})	2000 cycles (2 A g^{-1})	[S12]
**3.2 mg cm^{-2}**	0.5 M $Zn(TFSI)_2$ in AN	196 mAh g^{-1} (14.4 mA g^{-1})	120 cycles (14.4 mA g^{-1})	[S13]
**5-7 mg cm^{-2}**	1 M $ZnSO_4$ in H_2O	260 mAh g^{-1} (2400 mA g^{-1})	1000 cycles (2400 mA g^{-1})	[S14]
**0.9-1.2 mg cm^{-2}**	2 M $ZnSO_4$ in H_2O	470 mAh g^{-1} (500 mA g^{-1})	1000 cycles (10 A g^{-1})	[S15]
**1.0 mg cm^{-2}**	Ti_3C_2TX MXene + 2 M $ZnSO_4$ in H_2O	390 mAh g^{-1} (200 mA g^{-1})	300 cycles (1 A g^{-1})	[S3]

Supplementary References

[S1] F. Wang, O. Borodin, T. Gao, X. Fan, W. Sun et al., Highly reversible zinc metal anode for aqueous batteries. Nat. Mater. **17**(6), 543-549 (2018). https://doi.org/10.1038/s41563-018-0063-z

[S2] N. Zhang, F. Cheng, Y. Liu, Q. Zhao, K. Lei et al., Cation-deficient spinel $ZnMn_2O_4$ cathode in Zn $(CF_3SO_3)_2$ electrolyte for rechargeable aqueous Zn-ion battery. J. Am. Chem. Soc. **138**(39), 12894-12901 (2016). https://doi.org/10.1021/jacs.6b05958

[S3] C. Sun, C. Wu, X. Gu, C. Wang, Q. Wang, Interface engineering via $Ti_3C_2T_X$ MXene electrolyte additive toward dendrite-free zinc deposition. Nano-Micro Lett. **13**, 89 (2021). https://doi.org/10.1007/s40820-021-00612-8

[S4] P. Sun, L. Ma, W. Zhou, M. Qiu, Z. Wang et al., Simultaneous regulation on solvation shell and electrode interface for dendrite-free Zn ion batteries: achieved by a low-cost glucose additive. Angew. Chem. Int. Ed. **133**(33), 18395-18403 (2021). https://doi.org/10.1002/anie.202105756

[S5] L. Cao, D. Li, E. Hu, J. Xu, T. Deng et al., Solvation structure design for aqueous Zn metal batteries. J. Am. Chem. Soc. **142**(51), 21404-21409 (2020). https://doi.org/10.1021/jacs.0c09794

[S6] Q. Zhang, J. Luan, L. Fu, S. Wu, Y. Tang et al., The three-dimensional dendrite-free zinc anode on a copper mesh with a zinc-oriented polyacrylamide electrolyte additive. Angew. Chem. Int. Ed. **58**(44), 15841-15847 (2019). https://doi.org/10.1002/ange.201907830

[S7] W. Xu, K. Zhao, W. Huo, Y. Wang, G. Yao et al., Diethyl ether as self-healing electrolyte additive enabled long-life rechargeable aqueous zinc ion batteries. Nano Energy **62**, 275-281 (2019). https://doi.org/10.1016/j.nanoen.2019.05.042

[S8] W. Yang, X. Du, J. Zhao, Z. Chen, J. Li et al., Hydrated eutectic electrolytes with ligand-oriented solvation shells for long-cycling zinc-organic batteries. Joule **4**(7), 1557-1574 (2020). https://doi.org/10.1016/j.joule.2020.05.018

[S9] Y. Wang, N. Wang, X. Dong, B. Wang, Z. Guo et al., Zinc-organic battery with a wide operation-temperature window from -70 to 150 °C. Angew. Chem. Int. Ed. **59**(34), 14577-14583 (2020). https://doi.org/10.1002/ange.202005603

[S10] M. Yan, P. He, Y. Chen, S. Wang, Q. Wei et al., Water-lubricated intercalation in $V_2O_5*nH_2O$ for high-capacity and high-rate aqueous rechargeable zinc batteries. Adv. Mater. **30**(1), 1703725 (2018). https://doi.org/10.1002/adma.201703725

[S11] J. Zhou, L. Shan, Z. Wu, X. Guo, G. Fang et al., Investigation of V_2O_5 as a low-cost rechargeable aqueous zinc ion battery cathode. Chem. Commun. **54**(35), 4457-4460 (2018). https://doi.org/10.1039/C8CC02250J

[S12] P. Hu, M. Yan, T. Zhu, X. Wang, X. Wei et al., Zn/V_2O_5 aqueous hybrid-ion battery with high voltage platform and long cycle life. ACS Appl. Mater. Interfaces **9**(49), 42717-42722 (2017). https://doi.org/10.1021/acsami.7b13110

[S13] P. Senguttuvan, S.D. Han, S. Kim, A.L. Lipson, S. Tepavcevic et al., A high power rechargeable nonaqueous multivalent Zn/V_2O_5 battery. Adv. Energy Mater. **6**(24), 1600826 (2016). https://doi.org/10.1002/aenm.201600826

[S14] D. Kundu, B.D. Adams, V. Duffort, S.H. Vajargah, L.F. Nazar, A high-capacity and long-life aqueous rechargeable zinc battery using a metal oxide intercalation cathode. Nat. Energy **1**(10), 16119 (2016). https://doi.org/10.1038/nenergy.2016.119

[S15] Y. Yang, Y. Tang, G. Fang, L. Shan, J. Guo et al., Li^+ intercalated $V_2O_5{\cdot}nH_2O$ with enlarged layer spacing and fast ion diffusion as an aqueous zinc-ion battery cathode. Energy Environ. Sci. **11**(11), 3157-3162 (2018). https://doi.org/10.1039/C8EE01651H
